# Torsion of a huge subserosal uterine leiomyoma: A challenging case of acute abdomen

**DOI:** 10.4102/sajr.v27i1.2641

**Published:** 2023-05-11

**Authors:** Mankirat S. Dhillon, Anju Garg, Apoorva Sehgal, Sangeeta Bhasin

**Affiliations:** 1 Department of Radiodiagnosis, Maulana Azad Medical College and Lok Nayak Hospital, New Delhi, India; 2 Department of Obstetrics and Gynaecology, Maulana Azad Medical College and Lok Nayak Hospital, New Delhi, India

**Keywords:** subserosal, uterine leiomyoma, fibroid, pedunculated fibroid, torsion, ultrasound, magnetic resonance imaging, acute abdomen

## Abstract

Torsion of a uterine leiomyoma is an extremely rare, albeit life-threatening surgical emergency. A 28-year-old woman presented with acute abdominal pain. Imaging revealed a torsed subserosal uterine leiomyoma which was managed surgically and the diagnosis confirmed intraoperatively as well as on histopathology.

**Contribution:**

While intraoperative findings remain the primary means of diagnosis, radiologists should be familiar with the potential imaging findings of leiomyoma torsion as timely intervention can greatly improve patient outcome.

## Introduction

Uterine leiomyomas are the most common tumours in women of the reproductive age group.^1^ Although the prevalence of leiomyomas is high, acute complications are quite rare. However, when they do occur, failure to recognise and deal with these acute complications expeditiously can lead to catastrophic outcomes.^2^ Torsion of a pedunculated subserosal leiomyoma is considered an acute surgical emergency due to the high risk of ischaemic gangrene and subsequent peritonitis. It is often diagnosed intraoperatively as its imaging findings are non-specific and overlap with the more commonly encountered leiomyoma degeneration, thus making its pre-operative diagnosis rather difficult.^3^ This case report describes the imaging findings in a case of a torsed, pedunculated, subserosal uterine leiomyoma.

## Case Report

A 28-year-old nulliparous woman was referred from gynaecology emergency with complaints of excruciating lower abdominal pain, vomiting and vaginal bleeding for 3 days with acute worsening over the last 1 day. On physical examination, the patient’s abdomen was distended with a firm and tender pelvic mass reaching just above the umbilicus. The patient’s vitals were stable and laboratory investigations revealed a microcytic, hypochromic anaemia. The rest of her laboratory results were within normal limits.

**FIGURE 1:**
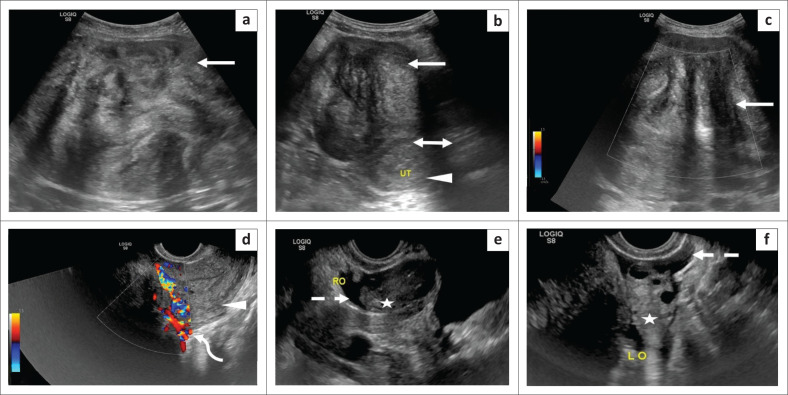
Grey-scale and Doppler ultrasound. Transverse (a) and sagittal (b) grey scale transabdominal ultrasound images of the abdomen and pelvis depict a large, well-circumscribed, heterogeneous, solid abdomino-pelvic mass (arrow), abutting the uterine fundus (double arrow in b) with no internal colour flow on Doppler evaluation (c). Transvaginal sagittal Doppler ultrasound image (d) demonstrates the vascular pedicle (curved arrow) between the uterus (arrowhead) and the mass, suggesting its uterine origin. Both ovaries were normal (⋆) and visualised separate from the mass with a small amount of anechoic free fluid in pelvis (dashed arrow) on transvaginal transverse (e) and sagittal grey scale images (f).

A transabdominal ultrasound (US) of the pelvis was performed which demonstrated a large, well-circumscribed, heterogeneous, hypoechoic, solid abdomino-pelvic mass, measuring 11 cm × 16 cm × 12 cm with posterior beam attenuation, abutting the uterine fundus. The mass showed no colour flow on Doppler evaluation. Transvaginal US revealed a vascular pedicle at the junction of the mass and the uterine fundus which showed absence of venous flow but presence of arterial flow on spectral Doppler. Both ovaries were identified separate from the mass and appeared normal. A small amount of anechoic free fluid was seen in the pelvis (Figure 1).

**FIGURE 2:**
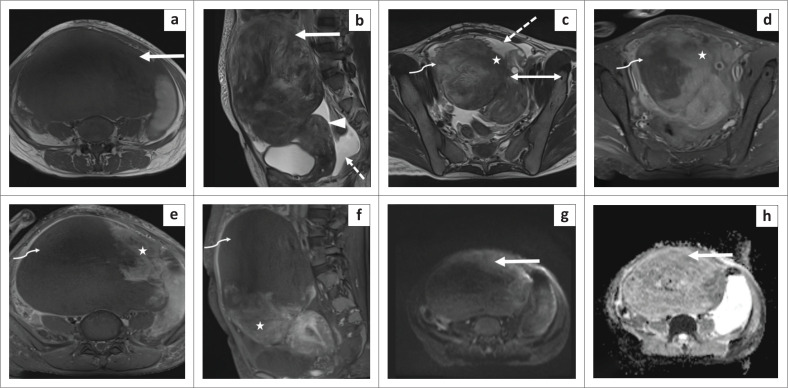
MR imaging in torsion of a subserosal uterine leiomyoma. MR images depict a large solid abdomino-pelvic mass lesion (arrow in a, b) continuous with the uterus (arrowhead in b) in the region of the left cornua (double arrow in c). The mass appears hypointense on the axial T1-weighted image (a) and heterogeneous on sagittal (b) and axial (c) T2-weighted MR images compared to the uterine myometrium. The majority of the mass appears heterogeneously hyperintense on the T2-weighted image (curved arrow in c) with a thin enhancing rim and non-enhancing core on post-contrast axial (d, e) and sagittal (f) MR images (curved arrow). A small juxtauterine area of relatively homogeneous T2 isointense signal (⋆ in c) and post-contrast enhancement (⋆ in d–f) is seen adjacent to its site of uterine attachment. The mass showed no diffusion restriction on diffusion weighted imaging (g) and the corresponding apparent diffusion coefficient map (h).

A provisional diagnosis of a subserosal uterine leiomyoma with possibilities of secondary torsion or degeneration were considered and contrast-enhanced MRI of the pelvis was performed for further evaluation. MRI confirmed the presence of a pedunculated, solid, abdomino-pelvic mass, continuous with the uterus in the region of the left cornua with flow voids at its site of the uterine attachment on T2-weighted images indicating the ‘bridging vessel sign’. The mass appeared isointense (to the myometrium) on T1 and heterogeneously hyperintense on T2-weighted images with no diffusion restriction. On the post-contrast images, the majority of the mass was non-enhancing with a thin enhancing rim and a small eccentric area of heterogeneous enhancement adjacent to its site of attachment with the uterus. A small amount of free fluid was seen in pelvis and both ovaries were unremarkable (Figure 2). Based on the clinical and imaging findings, a diagnosis of a torsed subserosal uterine leiomyoma was suggested.

The patient was counselled, written informed consent was obtained and exploratory laparotomy was performed. Intraoperatively, the mass was found to be a large subserosal leiomyoma attached to the uterus in the region of the left cornua by a narrow pedicle that was twisted around its axis. The mass was excised and subsequently sent for histopathological examination. Both ovaries and fallopian tubes were normal.

**FIGURE 3:**
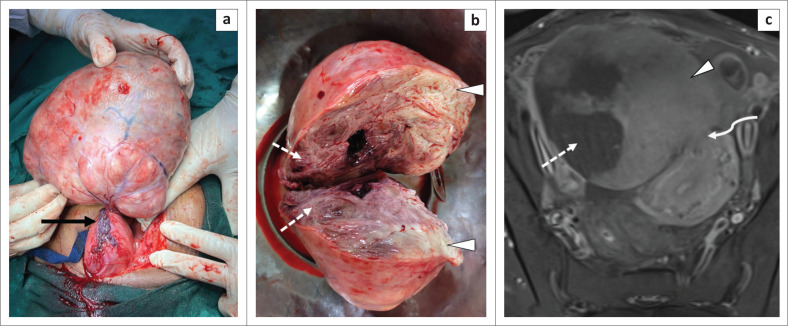
Gross specimen and cut section of the excised twisted subserosal leiomyoma. Intraoperatively, the mass was found to be a large subserosal uterine fibroid with a twisted vascular pedicle (arrow in a). Cut section of the gross specimen (b) revealed haemorrhagic necrosis in the majority of the mass (dashed arrows) which showed lack of contrast enhancement (dashed arrow) on the post-contrast MR image (c) with viable tissue at the site of uterine attachment which corresponded to the enhancement seen on the MR image (arrowhead in b, c). Figure (c) shows the uterine attachment site of the fibroid (pedicle indicated by a curved arrow).

On gross examination, the pedunculated uterine mass showed a twisted congested vascular pedicle with viable tissue adjacent to its pedicle corresponding to the enhancing component of the leiomyoma on MRI. On histopathology, the majority of the mass demonstrated necrosis with areas of internal haemorrhage, which corresponded to the non-enhancing component on MRI (Figure 3). The histopathogical results confirmed the diagnosis of a predominantly necrotic leiomyoma. The post-operative period was uneventful and the patient was symptom-free on follow-up.

## Discussion

Uterine fibroids or leiomyomas are benign, smooth-muscle neoplasms and are the most common gynaecologic tumours, found in 60% of women in their reproductive ages.^3^ Uncomplicated fibroids are often asymptomatic; however, when symptomatic they may result in abdominal pain, menorrhagia, dysmenorrhoea and infertility. Severe complications include torsion, degeneration, intraperitoneal haemorrhage, prolapse of submucosal fibroids, acute urinary retention and venous thrombosis.^2^

Torsion of a uterine leiomyoma is a rare cause of acute abdomen with an incidence of less than 0.25% reported in a single-centre retrospective study of complicated subserosal fibroids requiring surgery. Pedunculated subserosal fibroids carry the highest risk of torsion (as was seen in the presented case), particularly when the stalk is long and slender.^4^

Owing to its low incidence and overlapping clinical presentation with other causes of acute abdomen, this diagnosis is usually not considered pre-operatively. Even the imaging findings overlap with the more common leiomyoma degeneration, posing a diagnostic dilemma for the radiologist. This often leads to this uncommon condition being only diagnosed at the time of surgery.^3^

The clinical presentation is variable depending on the degree and speed at which the torsion develops. If the torsion is partial and intermittent with spontaneous untwisting, symptoms may be intermittent, constant or even resolve spontaneously. Complete torsion of the pedicle results in circulatory stasis that is initially venous with oedema and congestion followed by compression of the arterial blood supply that produces haemorrhagic necrosis and gangrene giving rise to excruciating pain as was seen in the presented case. If left untreated, haemorrhagic infarction of the torsed leiomyoma may lead to infection and peritonitis.^5^

Ultrasound is the primary imaging modality used to diagnose uterine leiomyomas, which appear as solid masses revealing a whorled appearance with posterior acoustic shadowing on grey-scale and a peripheral rim of vascularity on Doppler examination.^6^ Doppler interrogation can help identify the vascular pedicle in cases of subserosal leiomyomas (as depicted in Figure 1d in this case); however, the diagnosis of torsion still remains elusive. Some clinchers to the diagnosis of torsion include heterogeneity of the leiomyoma on grey scale, an abrupt cut off or twisting of its vascular pedicle and absent colour flow within the lesion even on power Doppler examination (as displayed in Figure 1c). However, these patterns are not regularly depicted.^4^ The congested pedicle in the presented case demonstrated an arterial waveform with absence of venous flow on Doppler. However, adnexal and uterine arterial waveforms can be normal on Doppler evaluation and this does not rule out torsion. Associated ascites may be seen.^5^ Thus, US has modest sensitivity and specificity not only in diagnosing torsion but also rarely in diagnosing subserosal leiomyomas, especially when the pedicle is thin. Ultrasound can help rule out other causes of acute abdomen which are more commonly encountered.^3^

In cases of inconclusive US findings, MRI is performed for its higher sensitivity and specificity. The vascular pedicle of a subserosal leiomyoma is better appreciated on MRI in the form of T2 flow voids at the interface between the uterus and the mass indicating the ‘bridging vessel sign’, suggesting a uterine origin of the mass and thereby differentiating it from an adnexal mass. In this case, the attachment site of the pedicle with the uterus was well appreciated on MRI (Figure 2c and Figure 3c). It is also important to identify normal ovaries separate from the mass.^7^ Uncomplicated leiomyomas appear homogeneously hypointense on T1 and T2 weighted images. In contrast, torsed leiomyomas undergo necrosis and gangrene thereby exhibiting heterogeneous hyperintensity on T2 weighted images and intermediate to hyperintense signal on T1 weighted images corresponding to ischaemia and haemorrhagic necrosis respectively. Diffusion restriction on diffusion weighted images can be seen (was not seen in this case) and a thin enhancing rim with lack of enhancement within the mass is seen on post-contrast images (as displayed in Figure 2d, e in this case).^6^ The non-enhancing core is consistent with necrosis while the regular rim of enhancement corresponds to obstructed peripheral veins with oedema.^8^ Ascites is an associated sensitive finding seen in the majority of the cases. The ‘dark fan sign’ is described as a fan-shaped area of poor contrast enhancement in the uterus adjacent to the torsed subserosal fibroid;^9^ however, this was not appreciated in this case.

CT has a limited role in the evaluation of leiomyomas; however, it may be performed when other causes of acute abdomen are suspected. CT findings include an interval increase in size of the leiomyoma, a lack of central enhancement with an enhancing rim, the ‘dark fan sign’ and ascites.^10^

Common differential diagnoses include leiomyoma with secondary degeneration and ovarian torsion. Degeneration in leiomyoma is more common than its torsion. It presents with acute pain; however, the clinical presentation is less sinister. It should be differentiated from torsion as emergency surgical intervention is not needed in cases of degeneration. Many types of fibroid degeneration are known to occur. In cystic degeneration, internal areas of fluid signal (hyperintense on T2 and hypointense on T1) are seen with a lack of enhancement on post-contrast images. Haemorrhagic degeneration can show internal T1 hyperintense areas which can be confused with areas of haemorrhagic infarction in a torsed necrotic fibroid. Fatty degeneration demonstrates fat signal intensity (hyperintense on T1 and intermediate to hyperintense on T2 weighted images) on MRI with suppression on fat saturated sequences. Hyaline degeneration appears similar to a non-degenerated myoma on T1- and T2-weighted scans; however, after the administration of contrast, hyaline degeneration displays decreased enhancement. Myxoid degeneration is more rare and shows marked hyperintensity on T2-weighted images with intense stromal enhancement and non-enhancement of the intervening mucinous lakes, the latter mimicking necrotic areas of a torsed fibroid.^11^Additionally, the presence of T2 hyperintense areas with diffusion restriction and low mean apparent diffusion coefficient value in a cellular leiomyoma and leiomyosarcoma can be confused with ischaemic necrosis in a torsed leiomyoma.^11^

An enlarged oedematous ovary showing peripherally displaced follicles with or without haemorrhagic infarction and a co-existent ovarian mass positioned in the midline and superiorly in front of the uterus is seen in ovarian torsion which may mimic a subserosal leiomyoma. The presence of an ovarian vascular pedicle is a valuable sign that helps in differentiating an ovarian mass from a subserosal uterine myoma.^12^ Other causes of acute pelvic pain such as endometriosis, pelvic inflammatory disease and appendicitis can easily be excluded with imaging.

Torsed uterine fibroids are managed surgically by myomectomy or hysterectomy depending on the age and desired fertility of the patient.^13^ The presented patient was in her reproductive age and, therefore, myomectomy was performed.

It is important to consider the possibility of torsed fibroid in the differential diagnosis in any woman presenting with an intra-abdominal and pelvic mass. It is also important to recognise the need for prompt exploratory surgery in any patient presenting with acute abdominal pain and the clinical suspicion of torsion. This case highlights the complexity of the diagnosis of torsed fibroid.

## Conclusion

While direct intraoperative visualisation of a twisted vascular pedicle remains the mainstay of diagnosis of a torsed, pedunculated, uterine leiomyoma, the presence of a non-enhancing subserosal fibroid with only a thin enhancing rim on imaging in the appropriate clinical setting should raise concern for torsion. However, this is often missed even by the most experienced radiologist due to rarity of the diagnosis and significant overlap in imaging findings.
